# Bottom Cooling During Culture Initiation Increases Survival and Reduces Hyperhydricity in Micropropagated Cannabis Plants

**DOI:** 10.3390/plants14060886

**Published:** 2025-03-12

**Authors:** Rambod Abiri, Declan O’Reilly, Andrew Maxwell Phineas Jones

**Affiliations:** Department of Plant Agriculture, University of Guelph, Guelph, ON N1G 2W1, Canada; rabiri@uoguelph.ca (R.A.); doreilly@uoguelph.ca (D.O.)

**Keywords:** cannabis, hyperhydricity, oxidative stress, micropropagation, bottom cooling

## Abstract

Hyperhydricity is characterized by morphological abnormalities and reduced plant vigour. This study investigated the use of a bottom cooling system (creating an approximate 2 °C temperature differential) during culture initiation to evaluate the impact on hyperhydricity in cannabis micropropagation. Nodal explants from two clonal triploid cultivars known to exhibit hyperhydricity, Higher Education 1 (HED-1) and Higher Education 2 (HED-2), were surface sterilized and placed in culture tubes using standard methods. Treatments included bottom cooling, metal pads without bottom cooling, and standard shelving (controls—no pad). Various morphological and physiological traits were assessed, including a detached leave water loss assay, dry mass, chlorophyll content, and survival rate. Plants cultured with bottom cooling showed significantly higher survival rates, healthier appearance, and improved physiological parameters compared to controls. In contrast, many control explants were hyperhydric with translucent and brittle leaves. Quantitative data revealed significant improvements in fresh weight (54.84% for HED-1 and 51.42% for HED-2), dry weight (36% for HED-1 and 8% for HED-2), chlorophyll fluorescence ratios (7.24% for HED-1 and 9.18% for HED-2), chlorophyll content (18.38% for HED-1 and 20.67% for HED-2), and cuticle/stomate function (30% for HED-1 and 27.27% for HED-2) using bottom cooling. Moreover, our morphological observation showed that almost 85% of control plants were hyperhydric, whereas only 10% of the plants cultured with a bottom cooling system were hyperhydric. This study confirmed that bottom cooling helps reduce the rate and impacts of hyperhydricity in cannabis and significantly improves the survival and quality of in vitro plants.

## 1. Introduction

*Cannabis sativa* L. (cannabis) is an important medicinal plant belonging to the Cannabaceae family that has recently gained significant global attention and economic impact [1]. The shift in regulations and legal status of cannabis in many regions has led to an increasing demand for large-scale propagation of insect and disease-free plants, driving the need to develop efficient micropropagation techniques [2,3]. Although vegetative cannabis production through stem cuttings is a reliable method for large-scale production [4], this method requires mother plants that occupy precious indoor facilities space to be maintained [5], and they can be exposed to various pest, pathogens, and viruses that can spread through vegetative propagation. To overcome these difficulties, micropropagation has been employed in the cannabis industry and research laboratories to facilitate the preservation of genetics in a safe and efficient manner, as well as large-scale propagation of insect, disease, and virus-free planting material [6,7]. Recent advancements in cannabis micropropagation techniques have significantly improved the efficiency and reliability of in vitro culture protocols [5,8,9,10,11,12], facilitating better research outcomes in gene transformation, genome editing, meristem culture, protoplast culture, and synthetic seeds [5,13,14,15,16,17,18,19]. However, early reports of micropropagation in cannabis have highlighted challenges related to long-term culture survival and low multiplication rates and specifically identified hyperhydricity as a common and major challenge. While there has been significant progress in cannabis micropropagation techniques in recent years, further improvements can be made and specific genotypes still present challenges for micropropagation [20].

Micropropagation is generally divided into the following five stages: stage 0: selection/maintenance of mother plants; stage 1: initiation of cultures; stage 2: multiplication of shoots/embryos; stage 3: shoot elongation and rooting; and stage 4: acclimatization [5]. Stage 1, initiation into tissue culture, is a pivotal phase in which the explant must transition from being photoautotrophic in the natural environment to mixotrophic growth in a sterile and much different setting. This comes with many challenges, including contamination and a propensity for hyperhydricity. Hyperhydricity is the most common physiological, morphological, and anatomical disorder in plant tissue culture. It is a complex disorder with a variety of symptoms, including hypolignification, poor cuticle development and stomate functioning, abnormal leaf morphology, poor root development and acclimatization, reduced chlorophyll content, and increased oxidative stress in the tissues [1,21,22,23,24,25,26,27]. However, the most diagnostic feature of hyperhydricity is the excessive accumulation of water within the apoplast, which results in the classic translucent, glassy appearance of the leaves that has led to the common terms of glassy or vitrified to refer to the disorder. As with the complex symptomology, many different contributing factors have been identified such as including insufficient light intensity, excess nitrates and sugars in the medium, the presence of PGRs, as well as high-humidity in vitro culture [28,29]. The complex nature of hyperhydricity has made it challenging both with respect to quantifying the degree of hyperhydricity in plants as well as developing methods to prevent it from happening or correct it once it has begun.

The first report on hyperhydricity dates back to 1964 when Phillips and Matthews [30] observed morphological malformations and physiological abnormalities during the tissue culture of carnations (*Dianthus caryophyllus*). Over time, this abnormality has been reported in more than 200 species of plant [26,31,32,33,34,35]. From a physiological perspective, hyperhydricity manifests through several indicators, including cellular hydration due to the accumulation of fluid between the intercellular spaces, chlorophyll deficiency, hypolignification, presence of starch granules within the plastid organelles, disruption of metabolic activity and alterations in protein synthesis and enzyme activity, enlarged intercellular spaces within the mesophyll, reduced cell adhesion, and diminished formation of the epicuticular layer on leaf surfaces [21,29,36,37,38,39,40]. Furthermore, hyperhydricity decreases the mitochondrial numbers, leading to the collapse of vascular tissue and damage to cellular structures and membrane integrity. This abnormality also results in heightened cell vacuolation and an expansion of intercellular spaces [40,41]. While leaf function, especially photosynthesis and gas exchange, is significantly affected by the occurrence of hyperhydricity, the disorder can damage roots and stems as well [22,29,40,42].

Since plant leaves constitute the initial layer of the plant’s response mechanism against stressors, the morphology and anatomy of leaves are mainly affected by the type and severity of environmental issues [43]. Furthermore, plant leaves are considered a physiological indicator to reflect the health, growth, and productivity of plants [44]. In cannabis tissue culture, hyperhydricity typically manifests in the form of translucent, deformed, and curled leaves accompanied by fragile shoots. Subsequently, these hyperhydric shoots have poor root development and low survival during the acclimatization process.

Various attempts have been made to overcome hyperhydricity-associated abnormalities, including optimizing culture media composition [20], the types and concentrations of gelling agents, adjusting pH levels and types of explants, designing vessels with improved gas exchange rates, and supplementing the media with silicate and calcium. For cannabis, Zarei et al. [45] showed that the addition of 4.86 mM exogenous potassium silicate (K_2_SiO_3_) to MS6 media reduced the nuFmber of translucent and malformed leaves. As a result, this treatment improved hyperhydricity symptoms and increased the survival rates of cannabis plants by up to 98%. Despite these efforts, the high incidence of hyperhydricity remains one of the most critical challenges during the initiation and multiplication phase of cannabis tissue culture [2,46,47,48,49,50,51].

Studies in other species have emphasized the significant impact of water potential (WP) and relative humidity (RH), which can be lowered using bottom cooling to mitigate hyperhydricity, but this has not been evaluated for cannabis [22,52,53]. It has been clearly shown that placing plant tissue culture vessels on a cooled pad results in the condensation of water vapour onto the medium rather than other surfaces (leaves, vessel walls, etc.), leading to a reduction in RH and leaf wetness [52]. Despite the positive response of plants to the bottom cooling system (BCS), the reduction in RH, and hyperhydricity, BCS may decrease the multiplication rate of some plants [52]. However, Piqueras [54] demonstrated that by decreasing hyperhydricity (up 35%), the multiplication rate was high in micropropagated carnation plants. Application of bottom cooling in micropropagated carnation reduced lipid peroxidation and relieved hyperhydric symptoms associated with oxidative stress [53]. Up to 80% of the reversion of hyperhydric plants to healthy plants has been reported in the carnation plantlets subjected to bottom cooling systems, suggesting that this could be a useful approach to address this challenge in cannabis micropropagation [54].

Culture initiation is a critical step in cannabis micropropagation that can have long-lasting impacts on the degree of hyperhydric growth. To date, the majority of efforts have focused on optimizing the media and plant growth regulator types and concentrations to enhance the efficiency of the initiation phase for cannabis plantlets. However, we hypothesized that environmental factors, in particular RH and leaf wetness, are larger contributing factors to this disorder and that bottom cooling will encourage healthier plant growth during and following the initiation phase. Ultimately, this will improve the efficiency of shoot growth, multiplication, rooting, and acclimatization. To the best of the authors’ knowledge, the current study is the first evaluation of bottom cooling for cannabis micropropagation.

## 2. Results

### 2.1. Qualitative Observation

**Figure 1:**
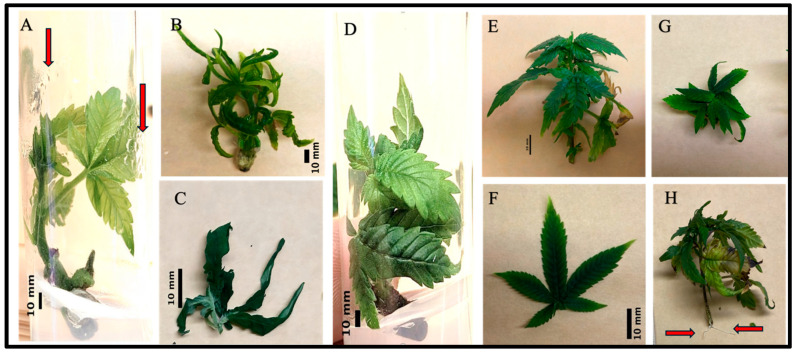
Morphological observations of cannabis plants were conducted on plants placed on a bottom cooling system, on pads without a cooling system, and without pads. (**A**) The image shows cannabis plants in test tubes after 30 days of culture in the control experiment and water droplets on the wall of sealed test tubes. (**B**) Several symptoms of hyperhydricity, including swollen stems, brittleness, a tendency to break, abnormal growth, reduced lignification, and excessive turgor pressure. (**C**) Hyperhydricity symptoms, such as a translucent or glassy appearance, brittleness, fragility, and thickened structure. (**D**) Healthy cannabis plants atop of bottom cooling system after 30 of culture in test tubes. (**E**) Healthy cannabis plants with flexible and resilient stems, uniform growth, optimal lignification, and moderate turgor pressure. (**F**) The appearance of certain symptoms on the leaves of cannabis, such as vibrant coloration, turgid texture, smooth surface, and even growth. (**G**) a sample of healthy plants taken from top angle of the bottom cooled plants, and (**H**) root induction sample from some healthy plants of bottom cooling system. Black bar shows 10 mm. Red arrows in (**A**) shows the water droplets due to hyperhydricity and red arrows in (**H**) represents the initial root induction of plants.

Overall, the rate and severity of hyperhydricity were greatly reduced by applying bottom cooling during the induction phase of cannabis. When cultured without bottom cooling, including those directly on the rack as well as those on the pads without hydronic cooling, condensation was commonly observed on all the inner surface of the vessel as well as on the leaves (the rate of observed condensation was 100% in the inner surface of non-bottom cooled plant’s vessels) (Figure 1A,B). In these conditions, both cultivars produced a large proportion of plants (≥85%) with hallmark signs of hyperhydricity. This included brittle and curled leaves with a translucent appearance and thickened shoots that were fragile and prone to breakage, sometimes without a recognizable structure. In contrast, there was a clear lack of condensation on the inner surface or plant tissues (the rate of observed condensation was almost absent on the inner surface of the bottom-cooled plant’s vessels) when placed on the hydronic bottom cooling system (Figure 1C). Almost all of the plants cultured (≃90%) with bottom cooling looked healthy with vibrant dark green coloration, firm and turgid shoots, more typical leaf morphology, less damaged tissues, and vigorous growth trajectory (Figure 1E–G). Further, roots were produced in the bottom-cooled plants more often (Figure 1H).

While the majority of plantlets cultured without bottom cooling exhibited symptoms of hyperhydricity (≥85%), some were healthy with normal growth rate and leaves shape (≃15%). Likewise, within the bottom cooled treatment, there were some signs of hyperhydric plants (≃10%), but at a greatly reduced rate. This observation highlights the complexity of plant responses within tissue culture systems, emphasizing that factors beyond the presence or absence of specific treatments can influence plant health and growth outcomes.

### 2.2. Detached Leaf Assay and Leaf Dry Biomass Assessment

**Figure 2:**
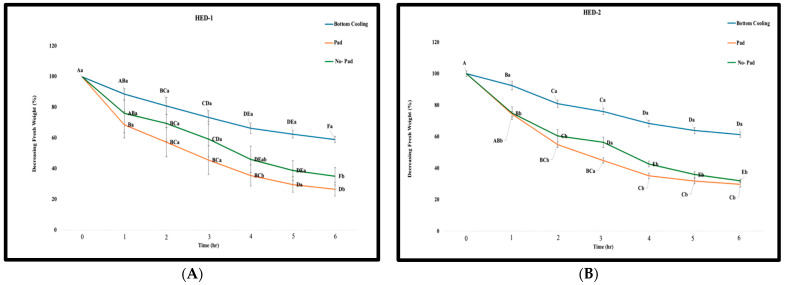
Decreasing leaf weight of (**A**) HED-1 and (**B**) HED-2 cultivars over 7 h in a detached leaf water loss assay. Note: Each point represents the mean value of three treatment groups, with error bars indicating the standard error (SE). Different letters indicate significant differences among accessions according to Duncan’s multiple range tests (*p* ≤ 0.05). Capital letters showcase the significant differences within each group, whereas small letters showcase the significant differences between 3 treatments.

Leaves produced by plants cultured with bottom cooling lost significantly less water and at a slower rate than both of the treatments performed without bottom cooling in the detached leaf assay. By the end of the assay, leaves from bottom-cooled plants had lost about 40% of their initial weight, while the control had lost around 60%. There were significant differences in the percentage of water lost at hours 4 and 6 among all three treatments for the HED-1 cultivar. There were significant differences in water loss at hours 1, 2, 4, 5, and 6 among all three treatments for HED-2 (Figure 2). Additionally, the results of the detached leaf assay showed that the rate of water loss was higher in the “no-pad” treatment (control 1) compared to the metal pad treatment without bottom cooling (control 2).

**Figure 3:**
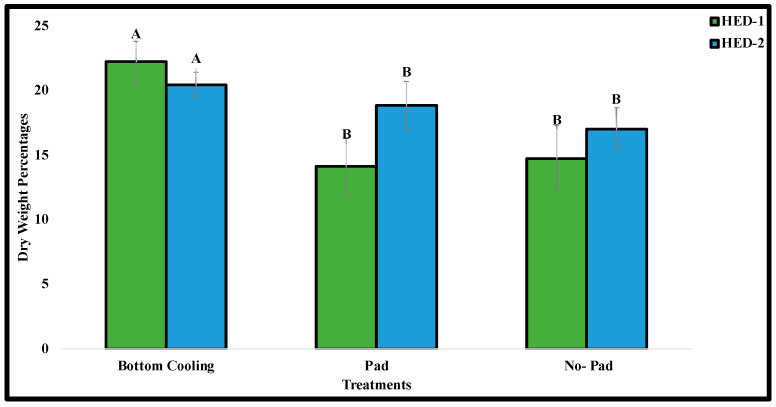
Dry weight percentage of ED-1 and HED-2 cultivars cultured with bottom cooling and without bottom cooling. (Pad and no pad treatments) Note: Bars represent the mean of three treatment groups with standard errors. Different letters indicate significant differences among accessions according to Duncan’s multiple range tests (*p* ≤ 0.05).

There were also significant differences in the percent dry weight between the bottom cooling treatments compared to control 1 and control 2 for both HED-1 and HED-2 cultivars. These results underscored that the bottom cooling treatment yielded the highest percent dry weight, while the two control treatments exhibited lower percent dry weights (Figure 3).

### 2.3. Chlorophyll Content

There were significant differences in both chlorophyll fluorescence ratio and chlorophyll content (*p* ≤ 0.05) between plants situated in the bottom cooling system and those in the control treatments for both HED-1 and HED-2 cultivars. The highest ratio of chlorophyll fluorescence ratio and chlorophyll content were observed in plants which were placed in the bottom cooling system for the HED-1 cultivar (Table 1). Our chlorophyll content assessment demonstrated that the chlorophyll fluorescence ratio for the HED-1 cultivar was 1.38 ± 0.057, 1.28 ± 0.054, and 1.20 ± 0.077, belonging to the plants placed atop of bottom cooling, pad, and non-pad systems, respectively (Table 1). Additionally, chlorophyll contents of the mentioned cultivar were 488.7 ± 35.16, 423.0 ± 35.13, and 374.7 ± 49.57 μg cm^−2^, belonging to the plants placed atop of bottom cooling, pad, and non-pad systems, respectively (Table 1). The result of the correlation coefficient between the chlorophyll fluorescence ratio and chlorophyll content in the HED-1 genotype was 0.999 (Table 1).

The highest ratio of chlorophyll fluorescence ratio and chlorophyll content were observed in the plants which were placed in the bottom cooling system for the HED-2 cultivar (Table 1). The SPAD analysis for the HED-2 cultivar showed that the chlorophyll fluorescence ratio for the plants a top of bottom cooling, metal pad without bottom cooling, and non-pad systems were 1.30 ± 0.028, 1.18 ± 0.059, and 1.13 ± 0.046, respectively (Table 1). Moreover, the chlorophyll contents were 441 ± 30.02, 369.33 ± 36.31, and 330.33 ± 30.75 μg cm^−2^ for the HED-2 plants a top of bottom cooling, pad, and non-pad systems, respectively (Table 1). The correlation coefficient of 0.998 between the Chlorophyll Fluorescence Ratio and Chlorophyll Content in the HED-2 genotype showed strong positive linear relations between the two variables (Table 1).

### 2.4. Quantitative Morphological Assessment

The survival rate for both cultivars was significantly higher in the bottom cooling treatment than either of the control treatments (Table 2). Furthermore, there were no significant differences observed between the survival rates of the two genotypes, whether they were subjected to a metal pad without bottom cooling or no-pad. The highest survival rate was observed for the plants which were in the bottom cooling treatment with 83.3% and 91.6%, for HED-1 and HED-2 genotypes, respectively. The survival rate for HED-1 remained consistent at 58.33% across both metal pads without bottom cooling and no-pad treatments. However, the survival rate of HED-2 remained consistent at 66.6% across both the pad and no-pad treatments (Table 2).

In this study, there were no significant differences among treatments for the HED-1 cultivar for leaf characteristics (Table 2). The highest number of leaflets, plant length, and length of the central leaflet were observed in the plants from the bottom cooling treatment with 7.0 ± 2.0, 10.4 ± 1.0, and 37.4 ± 0.4 (mm), respectively. However, the highest plant height belonged to plants placed in the magenta boxes on top of a pad (Table 2). Our data showed that the specific average number of leaflets was 7.0 ± 2.0, 6.0 ± 3.0, and 6.0 ± 1.0 for the HED-1 cultivar plants placed on the bottom cooling, pad, and non-pad systems, respectively (Table 2). Additionally, the average number of primary serrations of the central leaflet for the HED-1 cultivar were 10.4 ± 1.0, 8 ± 1.00, and 7 ± 3.00 for the plants placed atop of bottom cooling, pad, and non-pad systems, respectively (Table 2). Our plant length observations on the HED-1 cultivar demonstrated an average of 50.7 ± 4.7, 78.3 ± 3.6, and 68.02 ± 4.6 mm for the plants placed atop of bottom cooling, pad, and non-pad, respectively (Table 2). Moreover, the length of the central leaflets for the HED-1 cultivar were 37.4 ± 4.0, 24.2 ± 2.4, and 28.9 ± 1.7 mm for the plants placed atop of bottom cooling, metal pad without bottom cooling, and non-pad, respectively (Table 2).

In contrast, there were significant differences in terms of the number of leaflets between bottom cooling with both control treatments for HED-2. Nonetheless, there were no significant differences in terms of the number of primary serrations of the central leaflet and length of the central leaflets plant length for HED-2 (Table 2). For HED-2, the highest number of leaflets, number of primary serrations of the central leaflet, and length of the central leaflets were reported in the plants placed in the magenta boxes atop of bottom cooling system with 12 ± 1.0, 9 ± 2.0, and 34 ± 2.0, respectively (Table 2). Our study on the average number of some morphological traits of HED-2 cultivar in a top-of-bottom cooling system showed that the number of leaflets, number of primary serrations of the central leaflet, plant length, and length of the central leaflets were 12 ± 1.0, 9 ± 2.0, 53 ± 6.6 mm, and 34 ± 2.0 mm, receptively (Table 2). Furthermore, for the HED-2 cultivar, a top of the metal pad without bottom cooling, the average number of leaflets, the number of primary serrations of the central leaflet, plant length, and the length of the central leaflets were 8 ± 1.0, 7 ± 1.0, 52 ± 3.2 mm, and 22 ± 2.6 mm, respectively (Table 2). Finally, the average number of leaflets, number of primary serrations of the central leaflet, plant length, and length of the central leaflets for HED-2 cultivar a top of no-pad system were 7 ± 2.0, 8 ± 2.0, 52 ± 3.8 mm, and 25 ± 1.9 mm, respectively (Table 2). In addition to the mentioned traits, the measured fresh leaf weight varied across genotypes under different treatments. For example, the highest fresh leaf weight in the HED-1 cultivar was observed in plants under the pad and no-pad treatments, compared to those in the bottom cooling treatment. Conversely, the highest fresh leaf weight in the HED-2 cultivar was found in plants subjected to bottom cooling (Table 2).

## 3. Materials and Methods

### 3.1. Explants Resources, Disinfection Protocol, and Culture Condition

Nodal explants were obtained from two triploid clonal cultivars, Higher Education 1 (HED-1) and Higher Education 2 (HED-2) (Remix Genetics, Guelph, ON, Canada). 6-month-old mother plants were maintained as vegetative plants in an indoor growth facility in the Phytotron, University of Guelph [55]. The plants were grown in a Conviron GR192 growth chamber (North Dakota, USA) under long-day conditions (16/8 h). Plants were fertilized with a 17-5-17 NPK solution with an N concentration of 200 ppm. Lighting was provided by full spectrum LEDs with an average PPFD of 320 µmol/m^2^/s across the canopy. The average temperature was 22.7 °C, with daytime temperatures of 24–25 °C and nighttime temperatures of 19–20 °C. The average CO_2_ concentration was 1000 ppm, and average relative humidity of 60%.

Shoot explants with multiple nodes were first excised and rinsed for 5 min under running tap water. Subsequently, the explants were surface sterilized in a laminar flow hood for 10 min using a 10% commercial bleach (5.25% sodium hypochlorite, Clorox, Brampton, ON, Canada) and 0.1% tween 20, followed by three rinses of autoclaved distilled water, each lasting 5 min. The sterilized shoots were dissected into explants, each containing two nodes, and each explant was cultured into a test tube for 30 days in various treatment conditions. Next, 4 explants per block were sub-cultured together in GA7 vessels (Magenta LLC, Chicago, IL, USA). Culture tubes and magenta boxes all contained Driver and Kuniyaki Walnut (DKW) culture medium with vitamins (5.32 g/L, D2470, PhytoTech Labs, Lenexa, KS, USA) with 30 g/L sucrose, 1 mL/L Plant Preservative Mixture (PPM, Plant Cell Technology, Washington, DC, USA), and 6 g/L agar (A360-500; Fisher Scientific, Fair Lawn, NJ, USA). The pH of the medium was adjusted to 5.8 using 1.0 N NaOH before autoclaving for 20 min at 121 °C.

### 3.2. Bottom Cooling System

**Figure 4:**
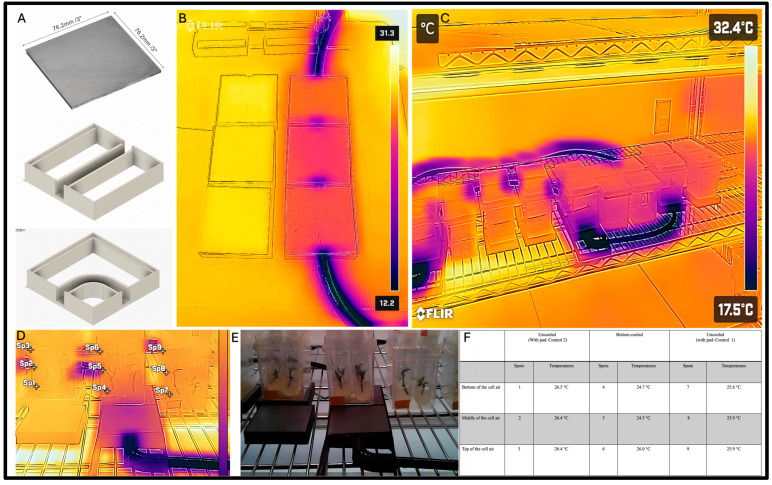
(**A**) Steel pads used for distribution of heat under magenta boxes and above coolant tubing, Fusion360 model of the straight plastic frame of the module used for bottom cooling, and Fusion360 model of the 90° turn plastic frame of the module used for bottom cooling. (**B**) The thermal photo of the direction of coolant flow through vinyl tubing taken by a FLIR One camera. (**C**) FLIR One thermal image of the implemented bottom cooling experiment. (**D**) A thermal photo and (**E**) the digital photo of a set of replications. (**F**) Shows the temperatures of different spots.

The experiment was run on a single shelf of a wire rack shelving (76.2 × 30.48 × 137.16 cm, Lulzbot, ND, USA) unit with full spectrum LED lighting providing a 16 h photoperiod with 40 ± 5 μmol m^−2^ s^−1^ light intensity. On this rack, there were 72 culture tubes (later transferred to 18 magenta boxes, each containing 4 cannabis plants) for a total of 72 plants. The cultures were placed on the cooling pad composed of 2 parts: a custom 3D printed plastic (PLA) frame and a metal heat dissipation shield. The plastic frame was designed using Fusion 360 and 3D printed with PLA on a Taz5 3D printer (Lulzbot, ND, USA). The frame was designed to house tubing in which the coolant was run through to serve as a hydronic bottom cooling system (Figure 4). The frame also contained edges to hold the metal pad that sat atop the frame. The metal pads were 7.62 × 7.62 cm welding coupons that helped distribute the temperature more evenly (Figure 4). These modules were arranged in a series, so an additional module was designed that houses a 90° turn for the tubing to better align the experiment (Figure 4).

The experiment consisted of three treatments arranged in a randomized complete block design (RCBD), including magenta boxes (1) placed on a metal pad connecting the bottom cooling system, (2) placed atop a metal pad without cooling, and (3) placed atop of normal shelves without metal pads. The vessels on a metal pad, which were not attached to the cooling system, were used as a second control treatment to account for any effect of the pad itself. Within each treatment, 8 culture tubes (later transferred into two magenta boxes) were incorporated, with four explants from each genotype. The total number of explants for each treatment was 12 explants of each genotype (four explants in each magenta box, three blocks), and each container was assumed as a replication for each plant.

Bottom cooling was achieved using a NESLAB RTE-9 (NESLAB Instruments, Portsmouth, NH, USA) endocal refrigerated circulating bath with temperature of −18 °C with a flow rate of approximately 15 L/min connected to ¼ inch inner diameter, ¾ inch outer diameter braided vinyl tubing (Onyx hose and tube inc., Guelph, ON, Canada). The NESLAB pump housed window washer fluid and recirculated it through the tubing to provide hydronic bottom cooling to the vessels [56]. After initial setup, the system was run for 24 h before temperatures were taken to let the coolant acclimate the modules and corresponding magenta boxes. Thermal images of the experiment were taken using a thermal camera (FLIR ONE) attached to an Android smartphone. Temperatures were measured weekly in the afternoon for each magenta box at three points: near the bottom, the middle, and near the lid of the magenta boxes.

### 3.3. Evaluation of Morpho- and Physiological Traits

The morpho- and physiological traits of cannabis plants were measured after 8 weeks of implementing the experiment using a combination of visual assessment and quantitative evaluation as follows:

### 3.4. Estimating Chlorophyll Content

To measure the chlorophyll fluorescence ratio (F735/F700) and chlorophyll contents, the central leaflet of each leaf was cut and placed between the sampled leaf clip with fibre installer of Opti-Science chlorophyl meter (CCM-330, Opti-Sciences Inc., Hudson, NY, USA) for small leaves.

### 3.5. Detached Leaf Water Loss Assay and Leaf Dry Biomass Assessment

Leaves of all cannabis plants in each vessel were harvested and placed on autoclaved papers under laminar flow hood. The rate of water loss was assessed by weighing all the leaves hourly over the course of 6 h.

Decreasing Leaf Relative Water Content = (Dehydrated fresh weight (hourly)/intial fresh weight (first read)) × 100

To determine the dry weight, the cut leaves were carefully enclosed in aluminum foil and subjected to desiccation within a Gravity Convection Laboratory Oven (Model: SGO1, Manufacturer SKU: SLG122, SLG122-EA, SLG, Randolph, MA, USA) set at 100 °C. The dry weight of the leaves was measured every 24 h, and the recording process was concluded when the dry weight of cannabis leaves remained constant, indicating that dehydration had ceased.

### 3.6. Morphological Assessment

Several morphological characteristics of treated and control cannabis plants, including survival rate, number of leaflets, number of primary serrations of the central leaflet, plant length (mm), and length of the central leaflet, were measured. The length-related traits were precisely measured using a Spurtar Vernier Caliper Measuring Tool 6” Stainless Steel (Guangzhou, China) to ensure accuracy in our data collection.

### 3.7. Statistical Analysis

The bottom cooling experiment was implemented based on a randomized complete block design (RCBD) with 3 replicates. The data were subjected to an analysis of variance (ANOVA) followed by Duncan’s multiple range test at *p* < 0.05 using SAS version 9.3. The morphological traits of the leaf were analyzed using ImageJ (version 4.3.2).

## 4. Discussion

Efficient propagation of cannabis can be achieved through various methods, including stem cutting, seed germination, and micropropagation. Stem cutting is a vegetative propagation technique and is the most reliable method for commercial propagation of the plant. However, this method encounters the risk of pathogen invasion and has the potential to rapidly spread pests and diseases [6,57,58,59]. Micropropagation of cannabis provides a high throughput alternative to cannabis stem cutting that can help prevent the spread of insects and diseases [60]. Despite the potential of micropropagation in cannabis, hyperhydricity frequently affects micropropagated plants, which is one of the biggest problems in establishing micropropagation systems in many species [61]. This condition results in malformations characterized by vitrescence, glauciness, translucency, and glassiness [62]. Hyperhydricity significantly reduces or even destroys the distinct morphology of palisade and spongy mesophyll tissues of leaves, resulting in a loss of mesophyll structure. [41]. The reported osmotic stress in hyperhydric plants may result from the accumulation of fluid (water) and the penetration of soft culture medium between the cells, leading to the creation of extensive intercellular spaces [29,39,63]. Additionally, drastic abnormalities are common in the apical meristem of hyperhydric plants, ultimately preventing strands of pro-cambium that extend downward in a basipetal direction from the leaf primordia from being developed. Reports show that fewer vascular bundles are produced in hyperhydric plants, and there is a lack of normal shoot arrangements. Proper uptake and transfer of water through the vascular system through evaporation forces is critical for normal growth in plants, which is impeded in hyperhydric tissues [23,64,65,66].

Generally speaking, alterations in the metabolic mechanisms of plants during hyperhydricity represent the plants’ response to environmental conditions. Analysis of the atmosphere within plant culture vessels confirms that hyperhydricity is strongly associated with the accumulation of gasses, primarily ethylene and CO_2_, along with high relative humidity [23]. Therefore, it can be inferred that several crucial factors associated with hyperhydricity include the temperature of vessels and cultivation conditions, genotype variations, age, type, and size of explants, concentrations of plant growth regulators, the composition of the medium, types, and concentrations of gelling agents, as well as the presence or absence of aeration [24,52,67,68,69,70,71,72,73,74].

Despite reports on the occurrence of hyperhydricity, there is a lack of clear documentation regarding the mechanisms underlying hyperhydricity in micropropagated plants. However, it has been widely confirmed that adverse conditions can increase the Reactive Oxygen Species (ROS) including organic hydroperoxide (ROOH), singlet oxygen (^1^O_2_), peroxy radical (ROO^•^), alkoxy radical (RO^•^), hydroxyl radical (OH^•^), hydrogen peroxide (H_2_O_2_), perhydroxy radical (HO_2_^•−^), superoxide (O_2_^•−^), and so forth [37,75,76,77,78,79,80]. Likewise, hyperhydricity generates H_2_O_2_ (placed in the intercellular spaces and the cell walls), OH, and O_2_^−1^ in mitochondria, chloroplast, apoplasts, and the cytosol [37,81], consequently resulting in abnormal processes of water-evaporation, -transportation, and -uptake [41]. In general, hyperhydric plants display a significant accumulation of water (fluid) within the apoplasts of leaves, causing gas exchange disruption [39] and, ultimately occurrence of hypoxia. Since hypoxia is strongly associated with oxidative stress, many of the malformations in the plant’s tissues could be attributed to oxidative stress [22,26]. As indicated, the heightened accumulation of ROS, stemming from the excessive uptake of water within the cells of the leaves, exerts deleterious effects on membrane enzymes, nucleic acids, lipids, and several other cellular structures. Observations confirmed the higher level of antioxidant enzyme activities (such as glutathione peroxidase, peroxidase, superoxide dismutase, and catalase) in the hyperhydric leaves [26,39,82,83]. Furthermore, direct interactions between ROS and ethylene metabolism have been reported in plants [84,85] subjected to environmental stress, as well as in hyperhydric plants [41].

Additional observations identify the role of imbalanced mineral levels, the influence of endogenous growth regulators, and the high accumulation of fluid with excessive moisture in the vessels as primary factors leading to the accumulation of ethylene and ROS, thereby causing malformation in plants under stress [41]. Hence, the downstream messenger, considered a secondary factor, is the activity of ROS. In plants with normal growth conditions, antioxidants can successfully eliminate ROS accumulation. However, if the antioxidant system fails to reduce ROS activity, high accumulation of ROS can lead to several cellular damages, particularly at the stomatal level. The irregular structure of stomata causes malfunction activity of the organ, leading to an imbalanced level of water absorption and losses in the plant’s tissue. Therefore, both the high accumulation of water and increasing levels of ROS can cause damage to the cellular structure, leading to hypoxia and hyperhydricity, respectively [23,40].

The occurrence and severity of hyperhydricity are related to many factors, including a variety of physical and chemical parameters [53]; however, one of the most important factors is high relative humidity (low vapour pressure deficit) that can cause plants to produce ethylene, which can accumulate in the vessels and further exacerbate the symptoms [86]. Bottom cooling systems have been employed to reduce evaporation and desiccation of the culture media, decrease relative humidity through condensation on the cooled vessel to encourage transpiration and reduce leaf wetness by providing a cooler surface for condensation to occur [56,87,88,89,90]. Various attempts have been made to find strategies to overcome hyperhydricity in vitro conditions in cannabis. For instance, using DKW supplemented with 0.5 µM TDZ decreased the hyperhydricity rate and produced broader and darker leaves in cannabis than plants cultivated in MS media with 0.5 µM TDZ [9]. Early studies showed that the content of macro-elements, including nitrogen, ammonium, and nitrate, have a significant effect on the physiological function, and anatomical and morphological structure of shoots in vitro [91,92]. The reports confirmed that decreasing the concentration of NH_4_NO_3_ improved shoot morphology and reduced hyperhydricity in *Aloe polyphylla* [93], *Phoenix dactylifera* [94], *Prunus avium*, and *Satix babylonica* [92]. Moreover, it has been shown that high concentration of hydrogen peroxide (more than 50 μM) increased the hyperhydricity rate in garlic shoots [51]. On the other hand, the application of osmotic stress-inducing agent like sodium chloride (NaCl—0.2%) in combination with polyethylene glycol (PEG—0.1%) could suppress the hyperhydricity rate during in vitro propagation of *Agave sisalana* [95]. However, our literature and previous experience showed that the application of one or more of the mentioned factors may not always effective to overcome to this abnormality. In addition, supplementing adjuvant materials (ex., silver ions) or increasing the concentration of some chemicals like PGRs may negatively influence the growth and development of the plants during in vitro culture. In contrast, our results emerged that application of bottom cooling can decrease the hyperhydricity by avoiding the plants to up-take excess water. Therefore, this system has high efficiency and less negative effect on the phycological function and morphological structure of the plant.

The higher percentage dry weight of plants grown on the bottom cooling system in our study suggests that hyperhydric plants contained significantly more water in their tissues, which is consistent with previous studies. This also aligns with previous findings from bottom cooling experiments conducted on carnation plants [22,53]. It is well-documented that hyperhydric potato shoots exhibit a lower dry weight compared to healthy plants [24]. It has been suggested that the increased dry weight observed in plants treated with bottom cooling systems could be attributed to a reduction in water uptake but may also result from a properly functioning evapotranspiration system which requires a vapour pressure deficit [53].

Chlorophyll content plays a critical role in plant health and the investigation of hyperhydricity in plants. It is well documented that the level of chlorophyll is generally lower in hyperhydric plants compared to healthy plants. For instance, it has been reported that the content of chlorophyll-a and chlorophyll-b were reduced in the hyperhydric gerbera (*Gerbera jamesonii* Bolus) plants. This decline leads to the appearance of pale green leaves in hyperhydric plants compared to the healthy plants [96]. Likewise, significant reductions have been reported in total chlorophyll, chlorophyll-a, and -b contents of hyperhydric Vanilla compared to healthy plants [25]. Moreover, lower chlorophyll content in hyperhydric carnation has been recorded [22]. Hyperhydric plants are also characterized by having fewer chloroplasts per cell and disruption of thylakoid structure and function [26].

In our experiment, the leaves of plants situated in both control treatments without bottom cooling were pale green, which is consistent with the lower levels of chlorophyll that were measured. In contrast, the healthy leaves of plants which were placed in the bottom cooled system were green with a firm texture and well-defined structure, which corresponds to the higher concentration of chlorophyll that was measured.

As reported in other studies, hyperhydric plants often have smaller stem diameters [97] and a lower size and frequency of stomata [96], along with reduced growth rates and delayed development [27,98]. These abnormalities can reduce the survival rate and differentiation abilities of hyperhydric plants. For instance, hyperhydric *Dendrobium officinale* (*D. officinale*) exhibited challenges in differentiation, proliferation, transplantation, and subculturing, resulting in a low survival rate. These restrictions stemmed from alterations in leaf size and stem diameter [27]. Furthermore, alterations in transpiration, photosynthesis, and the exchange of CO_2_ and O_2_ gasses in hyperhydric plants could lead to a reduction in survival rates [99].

In addition to the mentioned hypothesis, it can also be said that the abundance of ammonium ions is rapidly absorbed by plants from the media, leading to a rise in the utilization of carbohydrates and redirecting them away from the lignin metabolic pathway. Consequently, an imbalanced nitrogen-to-carbon ratio (with higher nitrogen accumulation) results in a deficiency of cellulose and lignin, leading to a decrease in cell wall thickness, an increase in water absorption, and ultimately, the manifestation of hyperhydricity [23,40].

Our experiment demonstrated that utilizing a bottom cooling system can reduce hyperhydricity by influencing stomatal conductance and transpiration rate, controlling water absorption and loss, and preventing high accumulation of water accumulation in cannabis leaves in the vessels. The mentioned regulation contributes to the prevention or reduction of hyperhydricity signs such as abnormal growth, necrosis, and tissue browning. Reportedly, the use of a cooled pad leads to a reduction in RH, condensation of water vapour in the medium, and a subsequent decrease in hyperhydricity [87,100,101]. Interestingly, it has been reported that leaflets exhibit larger sizes and lower RH when placed on the cooled pad, indicating normal growth activity and transpiration of healthy plants [100].

On a commercial scale, the bottom cooling system application can lead to more efficient and scalable production of plants during tissue culture. By maintaining optimal root zone temperatures, this technique reduces hyperhydricity and enhances the overall health of cannabis plants, resulting in higher success rates and improved yields in large-scale propagation efforts. Healthier cannabis plants with a low rate of occurrence of hyperhydricity are more likely to thrive and survive, producing a higher yield of robust cannabis plants suitable for large-scale cultivation. In fact, the application of a bottom cooling system may enhance the consistency and quality of propagated cannabis during tissue culture, aiming for large-scale production.

## 5. Conclusions

Hyperhydricity has been identified as one of the most common and important challenges in cannabis micropropagation. This study aimed to evaluate the potential of bottom cooling to address this challenge and improve micropropagation techniques. Our findings indicated that bottom cooling dramatically reduced hyperhydricity, increased plant survival rates, and generally improved plant health. The positive effects of bottom cooling were evident in terms of increased fresh and dry weight, enhanced chlorophyll fluorescence ratio, elevated chlorophyll content, and improved stomata structure in both cultivars. Although the results of this study underscored the significance of bottom cooling systems in inhibiting hyperhydric production under in vitro conditions, further research is warranted to determine the optimal temperature settings for these systems, investigating its effects on different cannabis genotypes, and/or exploring potential synergies with other micropropagation techniques. In addition, further studies should include optimizing cooling parameters for different cannabis genotypes, evaluating the impacts during multiplication and rooting, and investigating the long-term effects of bottom cooling on plant quality and yield.

## Figures and Tables

**Table 1:** Chlorophyll fluorescence ratio and chlorophyll content of both cultivars for bottom cooling, pad, and no-pad treatments.

	HED-1		HED-2	
	Chlorophyll Fluorescence Ratio	Chlorophyll Content (μg cm^−2^)	Chlorophyll Fluorescence Ratio	Chlorophyll Content (μg cm^−2^)
Bottom Cooling	1.38 ± 0.057 ^a^	488.7 ± 35.16 ^a^	1.30 ± 0.028 ^a^	441.0 ± 30.02 ^a^
Pad	1.28 ± 0.054 ^b^	423.0 ± 35.13 ^b^	1.18 ± 0.059 ^b^	369.3 ± 36.31 ^b^
No-Pad	1.20 ± 0.077 ^b^	374.7 ± 49.57 ^b^	1.13 ± 0.046 ^b^	330.3 ± 30.75 ^b^
Correlation coefficient	0.999		0.998	

Note: Different letters indicate significant differences among accessions according to Duncan’s multiple range tests (*p* ≤ 0.05).

**Table 2:** Quantitative morphological assessments of both cultivars.

	HED-1					HED-2				
	No. of Leaflets	No. of Primary Serrations of the Central Leaflet	Plant Length (mm)	Length of the C. L. (mm)	Survival Rate	No. of Leaflets	No. of Primary Serrations of the Central Leaflet	Plant Length (mm)	Length of the C.L. (mm)	Survival Rate
Bottom Cooling	7.0 ± 2.0 ^a^	10.4 ± 1.0 ^a^	50.7 ± 4.7 ^b^	37.4 ± 4.0 ^a^	83.33 ± 6.45 ^a^	12.0 ± 1.0 ^a^	9.00 ± 2.0 ^a^	52.7 ± 6.6 ^a^	34.4 ± 2.0 ^a^	91.66 ± 4.68 ^a^
Pad	6.0 ± 3.0 ^a^	8 ± 1.00 ^a^	78.3 ± 3.6 ^a^	24.2 ± 2.4 ^ab^	58.33 ± 5.76 ^b^	8 ± 1.00 ^b^	7 ± 1.00 ^a^	52.4 ± 3.2 ^a^	22.2 ± 2.6 ^a^	66.66 ± 3.43 ^b^
No-Pad	6.0 ± 1.0 ^a^	7 ± 3.00 ^a^	68.02 ± 4.6 ^ab^	28.9 ± 1.7 ^b^	58.33 ± 5.98 ^b^	7 ± 2.00 ^c^	8 ± 2.00 ^a^	52.01 ± 3.8 ^a^	25.3 ± 1.9 ^a^	66.66 ± 5.46 ^b^

Distinct letters imply significant distinctions between inoculation duration following Duncan’s multiple comparison test (*p* < 0.05). Note: C.L: central leaflet.

## Data Availability

Data generated or analyzed during this study are available from the corresponding author upon reasonable request.
